# MicroRNA fingerprints in juvenile myelomonocytic leukemia (JMML) identified miR-150-5p as a tumor suppressor and potential target for treatment

**DOI:** 10.18632/oncotarget.10577

**Published:** 2016-07-13

**Authors:** Pier Paolo Leoncini, Alice Bertaina, Dimitrios Papaioannou, Christian Flotho, Riccardo Masetti, Silvia Bresolin, Giuseppe Menna, Nicola Santoro, Marco Zecca, Giuseppe Basso, Giovanni Nigita, Dario Veneziano, Sara Pagotto, Katia D'Ovidio, Rossella Rota, Adrienne Dorrance, Carlo M. Croce, Charlotte Niemeyer, Franco Locatelli, Ramiro Garzon

**Affiliations:** ^1^ Department of Pediatric Hematology and Oncology, Istituto di Ricovero e Cura a Carattere Scientifico (IRCCS) Bambino Gesù Children's Hospital, Rome, Italy; ^2^ Division of Hematology, Arthur G. James Comprehensive Cancer Center, The Ohio State University, Columbus, OH, USA; ^3^ Department of Pediatrics and Adolescent Medicine, Division of Pediatric Hematology and Oncology, University of Freiburg, Freiburg, Germany; ^4^ Department of Pediatrics, “Lalla Seràgnoli” Hematology-Oncology Unit, University of Bologna, Bologna, Italy; ^5^ Department of Woman and Child Health, Haemato-Oncology Division, University of Padova, Azienda Ospedaliera di Padova, Padova, Italy; ^6^ Department of Paediatric Haemato-Oncology, Santobono-Pausilipon Hospital, Napoli, Italy; ^7^ Department of Paediatrics, Paediatric Unit 'F. Vecchio', University of Bari, Bari, Italy; ^8^ Department of Paediatric Haematology and Oncology, Fondazione IRCCS Policlinico S. Matteo, University of Pavia, Pavia, Italy; ^9^ Department of Molecular Virology, Immunology and Medical Genetics, The Ohio State University, Columbus, OH, USA; ^10^ Unit of General Pathology, Center of Excellence on Aging and Translational Medicine (CeSI-MeT), G. d'Annunzio University, Chieti, Italy; ^11^ Department of Medical, Oral and Biotechnological Sciences, G. d'Annunzio University, Chieti, Italy

**Keywords:** JMML, STAT5b, miR-150, microRNA, GM-CSF

## Abstract

Juvenile myelomonocytic leukemia (JMML) is an aggressive leukemia of early childhood characterized by aberrant proliferation of myelomonocytic cells and hypersensitivity to GM-CSF stimulation. Mutually exclusive mutations in the RAS/ERK pathway genes such as *PTPN11*, *NRAS*, *KRAS*, *CBL*, or *NF1* are found in ~90% of the cases. These mutations give rise to disease at least in part by activating STAT5 through phosphorylation and by promoting cell growth. MicroRNAs (miRs) are small non-coding RNAs that regulate gene expression, which are often deregulated in leukemia. However, little is known about their role in JMML. Here, we report distinctive miR expression signatures associated with the molecular subgroups of JMML. Among the downregulated miRs in JMML, miR-150-5p was found to target STAT5b, a gene which is often over-activated in JMML, and contributes to the characteristic aberrant signaling of this disorder. Moreover, loss of miR-150-5p and upregulation of STAT5b expression were also identified in a murine model of JMML. Ectopic overexpression of miR-150-5p in mononuclear cells from three JMML patients significantly decreased cell proliferation. Altogether, our data indicate that miR expression is deregulated in JMML and may play a role in the pathogenesis of this disorder by modulating key effectors of cytokine receptor pathways.

## INTRODUCTION

Juvenile myelomonocytic leukemia (JMML) is a rare and severe form of early childhood leukemia that is characterized by a clonal proliferation of myelomonocytic cells, progressive anemia, thrombocytopenia, hepatosplenomegaly and high fetal hemoglobin levels [1–3]. The prognosis of JMML is poor and, so far, the only curative treatment is allogeneic hematopoietic stem cell transplantation (HSCT). However, due to frequent disease relapse and treatment-related toxicity, the overall survival at 5 years after transplant ranges between 52 and 63% [3–6]. In addition, a small percentage of children do not have a suitable human leukocyte antigen matched donor. This highlights the urgent need to develop novel therapeutic approaches able to improve the current treatment results in JMML patients. Thus, to address this goal, the mechanisms of JMML leukemogenesis must be elucidated in order to be targeted pharmacologically.

JMML is caused by somatic and/or germline mutations on signaling effector genes of the RAS/ERK and JAK/STAT pathways, such as *NRAS, KRAS*, *PTPN11 (SHP2)*, *NF1* or *CBL*. These gain-of-function mutations are usually mutually exclusive and occur in approximately 90% of the cases [7–11]. Transformation of myelod progenitors by these mutations is associated with hyperactivation of the ERK, AKT [12], and Signal Transducer and Activator of Transcription 5 (STAT5) [13] pathways and hypersensitivity of myeloid precursors to granulocyte-macrophage colony-stimulating factor (GM-CSF) in culture [1, 14], which is considered a hallmark for this disorder.

MicroRNAs (miRs) are 18-24-nucleotide long non-coding RNA molecules that regulate gene expression by binding for the most part to the 3′ untranslated regions (3′UTRs) of their target genes, causing translation repression and in some cases target mRNA degradation [15]. MiRs play important regulatory roles in physiological cell processes, including cell cycle, apoptosis, differentiation and stem cell maintenance. Deregulation of miRs expression is a widely observed phenomenon in different type of cancers, including leukemia [16–18]. Elegant functional experiments have demonstrated that miRs are involved in leukemia initiation and progression [18, 19]. However, little is known about the role of miRs in JMML. A recent study identified the up-regulation of miR-223 and miR-15 in human induced pluripotent stem cells (hiPSCs) derived from *PTPN11* mutated JMML patients [20]. These results were validated in mononuclear cells obtained from *PTPN11* mutated JMML patients compared with controls. Blocking miR-223 functions in hiPSCs-derived myeloid cells normalized hematopoiesis. A second study reported that high levels of expression of the gene *LIN28b* correlated with higher hemoglobin F (HbF), low expression of let-7 miRs family members and with poor clinical outcome in JMML patients [21].

Based on these previous work and the well-described role of miRs in normal hematopoiesis and leukemias [18–21], we hypothesized that aberrant miRs expression may play a role in JMML pathogenesis. In order to address this question, we evaluated miR expression in a cohort of JMML patient's samples and controls, identified and validated deregulated miRs and then performed functional studies. Here we report miR signatures associated with JMML molecular subtypes and found that miR-150-5p, which expression is downregulated in JMML cases, targets STAT5 isoform b (STAT5b). Furthermore, we demonstrated that restoring miR-150-5p expression in JMML mononuclear cells decreases the sensitivity to GM-CSF. Altogether, our data suggest that the loss of miR-150-5p may contribute to the aberrant GM-CSF hypersensitivity observed in JMML patients.

## RESULTS

### MiR expression signatures associated with JMML

**Figure 1:**
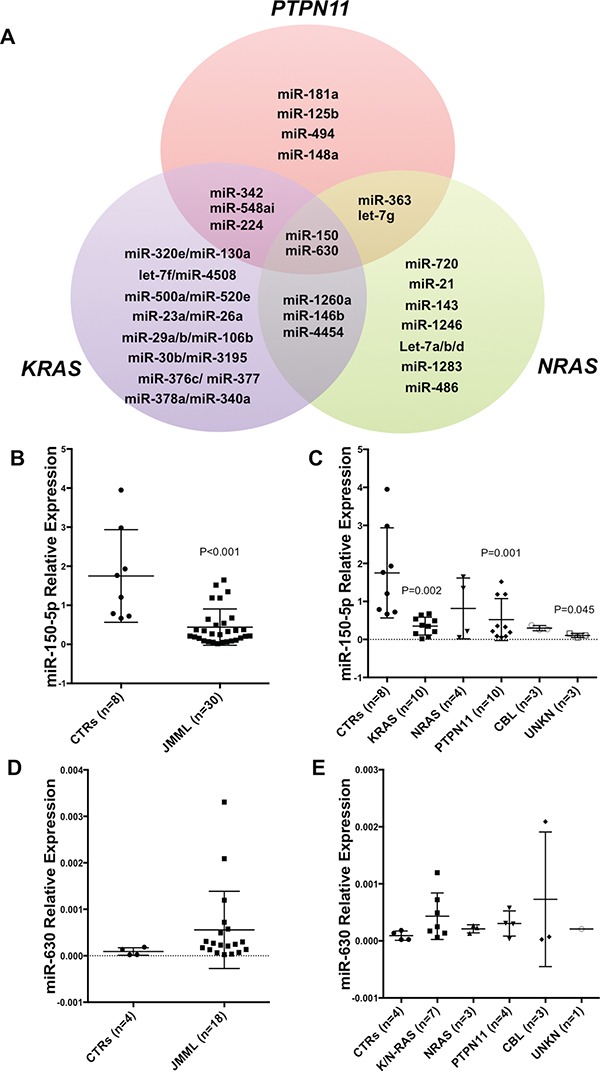
Deregulated microRNA Signature in JMML **A.** Venn diagram showing deregulated microRNAs (miRs) in JMML patients compared to controls. MiRs belonging to either *PTPN11*, *KRAS* and *NRAS* are shown in detail (See also Supplemental Table 1). CBL (n=3) and unknown patients (n=4) are not included due to low numbers of samples or unknown mutation status. **B.** Histograms showing miR-150-5p expression in a subset of JMML patients (n=30) vs controls (n=8) as measured by qRT-PCR (P<0.001). This subset of JMML patients was choosen based on the availability of RNA after the nCounter miR profiling. **C.** miR-150-5p expression according to the molecular subset of JMML and controls as measured by qRT-PCR (KRAS P=0.002; PTPN11 P=0.001; UNKN P=0.045). Results are shown as relative expression. **D.** MiR-630 expression in a subset of JMML patients (n=30) vs controls (n=8) as measured by qRT-PCR. **E.** MiR-630 expression according to the molecular subset of JMML and controls as measured by qRT-PCR. Results are shown as relative. P-values were calculated on three technical replicates using Unpaired two-tailed t-test. Unreported P-values are greater than 0.05 (P>0.05).

To identify miRs associated with JMML, we first analyzed the expression of miRs in a cohort of 40 newly diagnosed JMML patient samples and in 8 healthy controls (Table 1) using the nCounter technology (nanoString) as described in methods. First, we compared miR expression between all JMML cases (n=40) and the controls (n=8) and found 25 deregulated miRs; 11 were upregulated and 14 were downregulated in all JMML cases with respect to controls (P<0.05; Table 2). Subsequently, we grouped the JMML cases according to the most prevalent molecular subtypes in our cohort of patients (*PTPN11*, *KRAS* and *NRAS*) and compared each group individually with the controls. We identified distinctive miR signatures associated with the *PTPN11*, *KRAS* and *NRAS* molecular subtypes of JMML (Supplemental Table 1 and Figure 1A). The Venn diagram in Figure 1A shows the mutation-specific and common deregulated miRs among all the molecular subsets. Two miRs, miR-630 and miR-150-5p, are up- and down-regulated respectively, in all JMML molecular subtypes more than 2 fold, while miR-1260, miR-146b-5p and miR-4454 were downregulated in both *KRAS* and *NRAS* mutants only. MiR-224-5p and miR-548a were upregulated in both *PTPN11* and *KRAS* mutants, while miR-363-3p and let-7g-5p were up and downregulated in *PTPN11* and *NRAS* cases (Supplemental Table 1). Among the miRs downregulated in all JMML cases, but, in particular, in *KRAS* and *NRAS* mutated cases (Table 2 and Supplemental Table 1), there are several let-7 family members.

**Table 1:** JMML patients characteristics

Sample	Gender	Age (years)	Karyotype	Mutation
JMML 1	F	1.3	ND	CBL
JMML 2	F	6.2	normal	CBL
JMML 3	ND	0.6	normal	CBL
JMML 4	M	0.9	ND	KRAS
JMML 5	M	0.9	normal	KRAS
JMML 6	M	0.7	normal	KRAS
JMML 7	F	1.6	normal	KRAS
JMML 8	F	1.5	ND	KRAS
JMML 9	M	1.5	normal	KRAS
JMML 10	M	2.1	normal	KRAS
JMML 11	M	0.5	+mar	KRAS
JMML 12	M	0.1	normal	KRAS
JMML 13	M	0.5	−7, +mar	KRAS
JMML 14	M	0.3	−7, +mar	KRAS
JMML 15	M	0.9	normal	NRAS
JMML 16	F	1.7	normal	NRAS
JMML 17	F	1.7	normal	NRAS
JMML 18	M	1.0	normal	NRAS
JMML 19	M	1.1	normal	NRAS
JMML 20	ND	1.2	normal	NRAS
JMML 21	M	0.3	normal	NRAS
JMML 22	M	5.4	normal	NRAS
JMML 23	M	2.6	normal	NRAS
JMML 24	M	0.5	normal	NRAS
JMML 25	M	1.5	normal	PTPN11
JMML 26	F	5.7	−7, +21,+mar	PTPN11
JMML 27	M	ND	ND	PTPN11
JMML 28	F	2.4	normal	PTPN11
JMML 29	F	0.2	normal	PTPN11
JMML 30	F	3.7	ND	PTPN11
JMML 31	F	0.4	normal	PTPN11
JMML 32	M	6.2	normal	PTPN11
JMML 33	M	3.7	normal	PTPN11
JMML 34	F	2.0	ND	PTPN11
JMML 35	M	ND	ND	PTPN11
JMML 36	M	ND	ND	PTPN11
JMML 37	F	0.9	−7	UNKN
JMML 38	M	9.1	−7	UNKN
JMML 39	M	ND	normal	UNKN
JMML 40	M	2.6	normal	UNKN

ND= Not Determined; +mar= Marker Chromosome; +21= Chromosome 21 trisomy; −7= Chromosome 7 monosomy; normal= Normal Karyotype

**Table 2:** Deregulated microRNAs in JMML

Gene Name	Accession #	P-value	Fold-Change
***Upregulated***			
hsa-miR-630	MIMAT0003299	0.001	4.83
hsa-miR-3195	MIMAT0015079	0.014	2.70
hsa-miR-575	MIMAT0003240	0.014	2.69
hsa-miR-4508	MIMAT0019045	0.016	2.58
hsa-miR-224-5p	MIMAT0000281	0.024	2.34
hsa-miR-320e	MIMAT0015072	0.007	2.26
hsa-miR-494	MIMAT0002816	0.023	2.03
hsa-miR-548ai	MIMAT0018989	0.009	1.99
hsa-miR-222-3p	MIMAT0000279	0.018	1.69
hsa-miR-23a-3p	MIMAT0000078	0.040	1.61
hsa-miR-338-3p	MIMAT0000763	0.042	1.57
***Downregulated***			
hsa-miR-150-5p	MIMAT0000451	0.001	−5.21
hsa-let-7g-5p	MIMAT0000414	0.002	−3.16
hsa-miR-1260a	MIMAT0005911	0.009	−3.01
hsa-let-7a-5p	MIMAT0000062	0.010	−2.98
hsa-miR-4454	MIMAT0018976	0.021	−2.64
hsa-miR-148a-3p	MIMAT0000243	0.030	−2.31
hsa-miR-146b-5p	MIMAT0002809	0.009	−2.12
hsa-miR-342-3p	MIMAT0000753	0.010	−2.11
hsa-let-7f-5p	MIMAT0000067	0.021	−2.03
hsa-miR-26a-5p	MIMAT0000082	0.034	−2.01
hsa-let-7d-5p	MIMAT0000065	0.038	−2.01
hsa-miR-30b-5p	MIMAT0000420	0.019	−1.96
hsa-miR-29b-3p	MIMAT0000100	0.044	−1.94
hsa-miR-29a-3p	MIMAT0000086	0.024	−1.70

Significant deregulated microRNAs in JMML patients compared to Healthy Donors controls (P<0.05; see *miR expression data analysis* paragraph in Matherials and Methods section). Accession numbers from miRBase (http://www.mirbase.org) are reported for each miRNA.

Next, we validated the nCounter profiling results using qRT-PCR for the common deregulated miRs in all JMML subtypes: miR-630 and miR-150-5p. As shown in Figure 1, miR-150-5p was significantly downregulated in all JMML cases with respect to controls (Figure 1B; P<0.001). MiR-150-5p shows significant downregulation in all the different mutation subsets compared to controls, with the only relevant exception of *NRAS*-mutated patients (Figure 1C). MiR-630 expression was not significantly different in JMML cases with respect to controls (Figure 1D).

Since miR-150-5p has a critical role in hematopoiesis [22] and has been shown to act as a tumor suppressor in myeloid leukemias [23], we further investigated the functional role of this miR in JMML.

### MiR-150-5p targets STAT5b

To gain insight on potential miR-150-5p targets that may be relevant to JMML pathogenesis, we performed *in silico* target prediction analysis using miRWalk2 (http://zmf.umm.uni-heidelberg.de/apps/zmf/mirwalk2/documentation.html#use). Using this program, we identified 626 putative targets for miR-150-5p. Among these targets, STAT5b is a gene that, when deregulated, is involved in aberrant signaling and leukemogenesis [24]. Stat5b is a member of the Stat proteins family that is activated by tyrosine phosphorylation downstream of cytokine and growth factor receptors, including GM-CSF. Aberrant STAT5 activity induced by phosphorylation and/or increase expression has been shown to be closely connected to dysregulated GM-CSF signaling in JMML [13] and Chronic Myelomonocytic Leukemia (CMML) [14]. We hypothesized that the loss of miR-150-5p expression in JMML myeloid precursors may lead to the overexpression and consequent activation of its target Stat5b in the cytoplasm, contributing to GM-CSF hypersensitivity.

**Figure 2:**
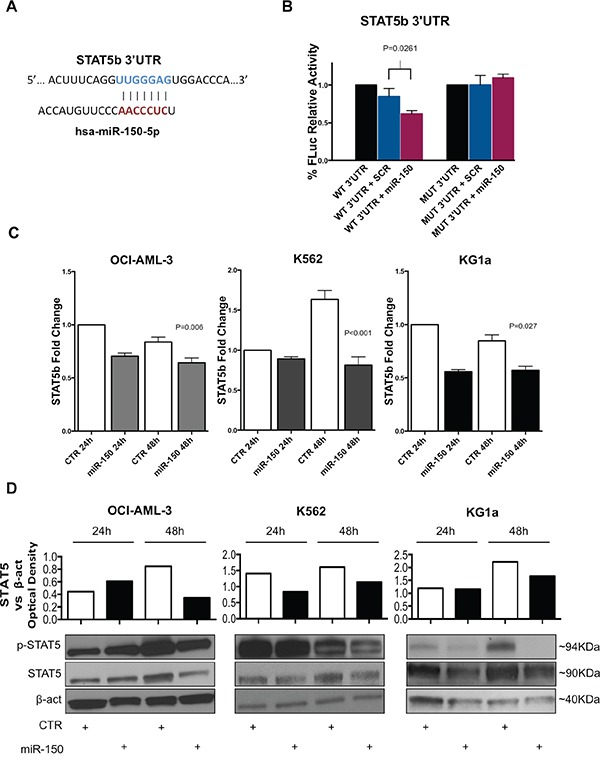
MiR-150-5p directly targets STAT5b **A.** Graph showing the interaction between the miR-150-5p seed sequence and the STAT5b 3′UTR. Pairing starts from 4462 to 4468 nuclotide (NM_012448; GenBank). **B.** Luciferase reporter assay for the interaction between STAT5b 3′UTR and miR-150-5p. Firefly Luciferase (FLuc) activities obtained from 293T cells co-transfected with wild type (WT) STAT5b 3′UTR construct and miR-150-5p mimic (P=0.026) or scrambled (SCR) oligonucleotides (control). Results are shown after normalization with Renilla Luciferaese. Mutant STAT5b 3′UTR contsructs (MUT) were also co-transfected with miR-150-5p mimic or SCR oligonucleotides. Results are representative of at least three independent experiments±SD. P-values were calculated using Unpaired two-tailed t-test. P-values greater than 0.05 (P>0.05) were not reported. **C.** STAT5b mRNA expression in OCI-AML-3 (P=0.006), K562 (P<0.001) and KG1a (P=0.027) cell lines after transfection with miR-150-5p or empty vector control (CTR) as measured by qRT-PCR. Results are shown as fold change in STAT5b mRNA expression with respect to control sample at 24 hours (CTR). **D.** Western Blotting analysis of Stat5, phosphorylated Stat5 (p-Stat5) and β-actin (loading control) expression in OCI-AML-3, K562 and KG1a cell lines after transfection with miR-150-5p or empty vector control (CTR). Densitometry plot shows STAT5 upregulation in cell lines. Optical density was calculated using β-actin as reference. Results are representative of at least two independent experiments. Densitometry histograms are relative to represented experiment below.

To validate this interaction, we used a luciferase reporter vector containing the 3′UTR sequence of STAT5b predicted to interact with miR-150-5p (Figure 2A) and co-transfected with miR-150-5p mimic or control (scrambled) into 293T cells. As shown in Figure 2b, asignificant reduction of luciferase activity was detected for STAT5b 3′UTRs in presence of miR-150-5p mimic with respect to controls. Furthermore, the observed luciferase reduction was abrogated when we co-transfected a luciferase reporter vector containing the mutated 3′UTR sequence of STAT5b along with the miR-150-5p mimic or controls (Figure 2B).

Next, we overexpressed miR-150-5p mimic or control oligonucleotides in three different AML cell lines (K562, OCI-AML-3 and KG1a) that do not express miR-150-5p, using nucleoporation as described in Methods, and measured STAT5b mRNA and protein expression. As shown in Figure 2C, we found a slight STAT5b mRNA downregulation after miR-150-5p overexpression in all the three cell lines (Supplemental Figure 1). However, Western Blotting analysis showed a significative decrease of Stat5 protein levels and phosphorylated Stat5 (p-Stat5) in all cell lines after 48 hours post transfection (Figure 2D and 2E).

### JMML murine model recapitulates miR-150-5p deregulation and STAT5 overexpression found in human patient samples

**Figure 3:**
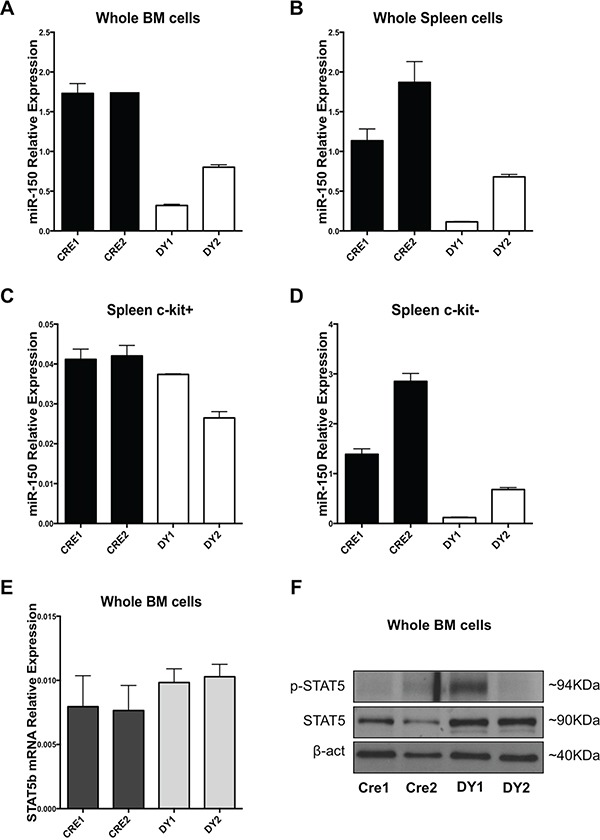
Bone marrow and splenic cells from a Ptpn11 mutated murine model resembling JMML exhibit miR-150-5p expression downregulation and STAT5b upregulation **A.** miR-150-5p expression in whole BM cells, **B.** unselected splenocytes and **C.** c-kit (CD117) positive or **D.** c-Kit negative selected splenocytes of Mx1-Cre; LSL-Ptpn11-D61Y (DY) mice and control mice (Mx1-Cre or CRE) as measured by qRT-PCR. Results are shown as relative expression after normalization with snoU6. **E.** STAT5b mRNA expression in whole BM cells from DY and CRE mice. **F.** Western Blotting analysis of Stat5, p-STAT5 and β-actin of whole BM cells from DY and CRE mice.

After validating STAT5b as *bona-fide* target of miR-150-5p, we attempt to demonstrate whether miR-150-5p, STAT5b mRNA and Stat5 protein were also deregulated in a murine model of JMML. To answer this question, we used a Ptpn11 mutated murine model of JMML (Mx1-Cre;LSL-Ptpn11-D61Y). These mice develop a fatal myeloproliferative disorder, featuring leukocytosis, anemia, hepatosplenomegaly, and factor-independent colony formation by BM and spleen cells [25]. We first assessed miR-150-5p expression by qRT-PCR in whole BM cells and splenocytes from two different Ptpn11-mutated mice and their respective controls (Mx1-Cre transgenic mice on a C57BL/6 background). MiR-150-5p was found downregulated in the two mouse samples, both in whole BM cells and splenocytes compared to controls (Figure 3A and 3B). We also evaluated miR-150-5p levels in earlier progenitor cells (c-kit (CD117) receptor positive selected cells) from mouse spleens and found downregulation of miR-150-5p in c-kit^+^ selected cells, as well as c-kit^−^ cell populations (Figure 3C and 3D). In addition, we measured STAT5b mRNA and Stat5 protein levels in these mouse samples. RT-qPCR results showed a mild increase at their mRNA level (Figure 3E), whereas Stat5 protein levels were clearly upregulated in the Ptpn11 mutated mice compared to controls (Figure 3F). p-Stat5 showed increase in only one of the two Ptpn11 mutated murine samples (Figure 3F).

### MiR-150-5p overexpression decreases hypersensitivity of JMML BM mononocuclear cells to GM-CSF

**Figure 4:**
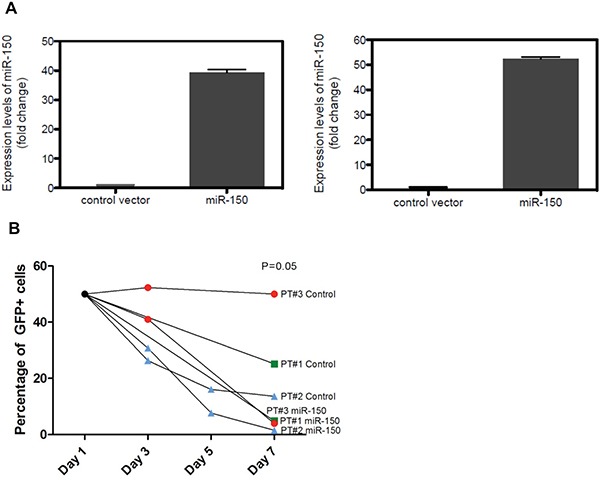
MiR-150-5p overexpression decreases hypersensitivity of JMML bone marrow (BM) mononuclear cells to GM-CSF **A.** MiR-150-5p expression levels in BM mononuclear cells from two JMML patients infected with miR-150-5p overexpressing lentivirus or empty vector (Control) evaluated by qRT-PCR. Expression levels of U44 were used to normalize the raw miR-150-5p values. **B.** *In vitro* proliferation assay of BM mononuclear cells from three newly diagnosed JMML patients after infection with miR-150-5p lentivirus or empty vector (control). After infection with GFP expressing constructs, GFP-sorted cells were mixed with non-GFP cells at a 4 to 1 ratio. GFP-positivity was evaluated by flow cytometry. The frequency of GFP positive cells in control versus miR-150-5p overexpressing JMML cell populations was calculated for a maximum period of 7 days after sorting the cells.

To assess the impact of miR-150-5p expression on JMML cells sensitivity to GM-CSF, mononuclear cells from the BM of three JMML patients were transduced with lentiviral particles containing either empty control vector or miR-150-5p overexpressing GFP+ vector. GFP+ sorted cells were cultured with GFP- cells at established ratios in the presence of cytokines, including GM-CSF as described in methods and the frequency of GFP+ cells was measured overtime. Successful overxepression of miR-150-5p was demonstrated by qRT-PCR (Figure 4A). As shown in Figure 4B, overexpression of miR-150-5p decreased significantly cell proliferation with respect to empty vector.

## DISCUSSION

The prognosis of JMML is still poor and the only potentially curative treatment is allogeneic HSCT [6, 7]. However, the overall survival after allogeneic HSCT is about 52 to 63% [3–6]. Thus, novel approaches are needed to improve patient outcomes after transplantation and to control the disease in those children who do not have an immediately available donor. While most of the mutations in JMML have already been discovered and affect genes involved in cytokine signaling such as RAS/ERK and JAK/STAT pathways [7–11], the role of non-coding RNAs in this disease is still undefined. Here, we aimed to identify miR signatures associated with JMML and to dissect their functional role in the pathogenesis of the disease with the hope that novel targets for therapeutic intervention could be discovered.

To achieve this goal, we profiled a large number of JMML samples, considering the rarity of the disease, using the nCounter miR platform. We identified miR signatures that differentiate JMML molecular subtypes from controls. Among the downregulated miRs in JMML patients, we found several let-7 family members. These results are consistent with the recently published manuscript from Helsmoortel et al [21], where several let-7 family members were found at reduced levels in *LIN28B* overexpressing JMML cases. *LIN28b* is known to inhibit let-7 family members [26] and may explain at least in part the down-regulation observed of let-7 in JMML. In addition, as described previously, let-7 family members directly recognize and bind to the 3′UTR of *KRAS* and *NRAS* blocking RAS translation [27–28]. The finding of miRs that negatively regulates RAS signaling in JMML cases is relevant due to the role of aberrant RAS signaling in JMML. Remarkably, both *NRAS* and *KRAS* have let-7-complementary sites in their 3′UTRs and have been validated as let-7 targets in several malignancies [27, 28]. Overexpression of let-7 in cancer cell lines, primary cancer samples and animal models of RAS induced cancers represses the expression of the K-Ras and N-Ras proteins resulting in profound antitumoral effects [29–31]. Moreover, GM-CSF hypersensitivity of myeloid progenitors is caused by a selective inability to downregulate RAS-dependent signalling pathways [14]. This observation is supported by the different mutations that affect the RAS pathways (*KRAS, NRAS).* Thus, it is possible that the loss of let-7 family members expression further contributes to unleash RAS signaling in JMML.

We further investigated miR-150-5p and miR-630, which were deregulated to a greater extent in all molecular subtypes of JMML as compared to controls (Figure 1A). We validated the global downregulation of miR-150-5p but not of miR-630 in all JMML patient samples with respect to controls using a different method of miR detection (qRT-PCR). After validation, we focused our studies on miR-150-5p and aimed to identify potential targets of this miR and to provide functional insights. MiR-150-5p is localized on chromosome 19q13 and plays a crucial role in cell fate decisions between erythroid and megakaryocytic differentiation and in controlling B-cell differentiation [19, 22]. The expression of miR-150-5p is deregulated in various solid cancers, such as lung, gastric, colorectal, breast and pancreatic cancer [32]. In leukemias, miR-150-5p act as a tumor suppressor in *MLL*-rearranged and other subtypes of acute myeloid leukemia (AML), by targeting *HOXA9* and *MEIS1,* either directly or indirectly [23, 33]. Remarkably, it has been reported that MLL in combination with *MYC* and *LIN28B* downregulates miR-150-5p expression in AML [23]. This is relevant to JMML, since *LIN28B* has been reported to be upregulated in these patients and may be one of the factors involved in the downregulation of miR-150-5p in this disease [21].

Here, we show that STAT5b is a *bona fide* target of miR-150-5p. STAT5 is a well-known master regulator of early myeloid proliferation and differentiation [34–36] and aberrant STAT5 activity induced by phosporylation and/or increase expression has been shown to be closely associated with dysregulated GM-CSF signaling in JMML [13] and CMML [14]. The ectopic overexpression of miR-150-5p in myeloid cell lines decreases both STAT5b mRNA and Stat5 protein level.

In a Ptpn11-mutated animal model that recapitulates a myeloproliferative condition resembling JMML [25], we found STAT5b mRNA and Stat5 protein upregulation together with miR-150-5p downregulation in whole BM cells, splenocytes and c-kit+ cells. Last, the ectopic overexpression of miR-150-5p in mononuclear cells from JMML patients blocks the GM-CSF effects on cell proliferation. Altogether, our results indicate that deregulated miR-150-5p may play a role in the upregulation of key components of the GM-CSF signaling pathways, thus contributing to JMML pathogenesis. The increased availability of the substrate (STAT5b) caused by downregulation of miR-150-5p, may enhance the phosphorylation activation of STAT5b caused by the gain of function mutations in the RAS/ERK pathway key genes, such as *KRAS, NRAS, NF1 and PTPN11* in JMML.

Despite the clinical significance of targeting RAS in JMML, as well as in other cancers, no drugs that target directly RAS have been developed so far. There are many reasons for this, including the lack of suitable pockets in the molecule surface for small molecule inhibitors [37–39]. Thus, our results have therapeutic implications, since they open the door to use miR therapeutics to target JMML aberrant signaling that is caused by known mutations. We have shown that miR-150 overexpression dampens STAT5b expression and activity and decreases significantly the cell proliferation of primary JMML samples. Second, overexpressing let-7 family members in JMML patients may target RAS directly and induce profound antileukemic effects as it has been shown for other RAS-dependent malignancies [29–31]. Clinical therapeutic trials in cancer are now being conducted using miR-based therapies [40]. Therefore, it is possible to envisage the use of such a strategy for JMML in the near future.

Taken together, our results identify miR-150-5p downregulation in JMML patients and in a JMML animal model. Functionally, miR-150-5p directly inhibits the translation of STAT5b mRNA. The loss of miR-150-5p in JMML may increase the availability of Stat5b to undergo phosphorylation and activation, which may further enhance the GM-CSF sensitivity caused from gain of function mutations. Restoration of miR-150-5p levels could be a novel approach to target the aberrant signaling in JMML.

## MATERIALS AND METHODS

### Patient samples

Thirty-five frozen bone marrow (BM) and five peripheral blood (PB) samples from 40 newly diagnosed JMML patients were obtained from the Ospedale Pediatrico Bambino Gesù (OPBG) Rome, the European Working Group on Myelodisplasia (MDS) in Childhood (EWOG-MDS) and the Department of Pediatrics, University of Padova. Eight frozen BM samples from healthy children (unused samples from matched sibling donor transplants) were also obtained from the tissue bank at OPBG. Informed consent was obtained from either parents or legal guardians according to the Declaration of Helsinki. Approvals for this study were obtained from the Institutional Review Boards (IRB) of each institution. Mononuclear cells (MNC) were isolated by density gradient centrifugation, diluted in 90% fetal bovine serum (FBS) plus 10% dimethyl sulfoxide (DMSO) and stored in liquid nitrogen.

### Cell Lines and miR-150-5p overexpression

OCI-AML-3 (ACC-582; DSMZ), K562 (CCL-243; ATCC) and KG1a (CCL-246.1; ATCC) cell lines were cultured in RPMI-1640 (Corning Cellgro, Corning, NY, USA), supplemented with either 10% FBS (OCI-AML-3 and K562) or 20% FBS (KG1a) and 1% Penicillin/Streptomycin. Cells were seeded at 2×10^5^ cells/mL initial concentration. MiR-150-5p overexpression was obtained using AMAXA Nucleofector (Lonza, Basel, Switzerland) using Kit V, Kit L and Kit T for K562, KG1a and OCI-AML-3 respectively, transfecting 2.5μg of pEZX-MR04 miRNA precursor plasmid (Genecopoeia, Rockville, MD, USA) for each condition carrying full length miR-150-5p precursor clone, following manufacturer's instructions. After transfection, cells were seeded in 6 multi-well plates at the concentration of 10^6^ cells/mL in each well. Efficiency of overexpression was assessed 48 hours post transfection by qRT-PCR.

### Murine BM and splenocytes purification and KIT+ (CD117+) cell selection

Femurs, tibias and whole spleens of Ptpn11 mutated mice (Mx1-Cre; LSL-Ptpn11-D61Y) and controls (Mx1-Cre) were kindly provided by Dr. Benjamin Neel from New York University School of Medicine, Perlmutter Cancer Center (New York City, NY, USA). BM was flushed out from femurs and tibias in sterile phosphate buffered saline (PBS), centrifuged at 350 RPM for 10 minutes, and red blood cells were lysed with ammonium chloride (NH_3_Cl) solution from Stem Cell Technologies (cat. number #07850). Whole spleens were processed by mechanical homogenization and filtered to avoid presence of clumps in the solution. Red blood cells were lysed as above. C-kit+ cells were separated using CD117 positive selection kit from Miltenyi Biotec (Bergisch Gladbach, Germany) (cat. number #130-091-224), following manufacturer instructions.

### RNA purification and quality control

Total RNA was extracted using TRIzol (Invitrogen, Carlsbad, CA, USA) and purified using RNA Cleanup and Concentration kit (Norgen, Thorold, ON, Canada). RNA quantification was performed using Nanodrop 2000 (Thermo-Scientific, Waltham, MA, USA) and RNA integrity and purity was assessed with RNA Bioanalyzer kit (Agilent Technologies, Santa Clara, CA, USA) considering samples with an RNA Integrity Number (RIN) at least of 7.

### miR expression data analysis

MiR expression profile of mononuclear cells from 40 newly diagnosed JMML patients and 8 healthy controls was performed using the nCounter Human v2 miRNA Expression Assay loading 100 ng of total RNA for each patient and control according to manufacturer's protocol (nanoString Technologies Seattle, WA, USA; Complete list of analyzed microRNAs in Supplemental Table 2). Normalization was performed using the nSolver Analysis Software (nanoString Technologies), applying the geometric mean of the top 100 miRs in all samples, as recommended by NanoString. P-values were calculated using the LIMMA package from the Bioconductor R project, as provided by the Carmaweb tool [41]. The p-values were adjusted for multiple testing using the Benjamini and Hochberg method to control the False Discovery Rate (FDR). Venn diagram was generated using free online software (http://www.educationalworld.com).

### Quantitative RT-PCR

ImProm-II™ Reverse Transcription System (Promega, Fitchburg, WI, USA) or TaqMan MicroRNA Reverse Transcription Kit (Life Technologies, Carlsbad, CA, USA) was used for gene and miR reverse transcription, respectively. Quantitative RT-PCR (q-RT-PCR) was performed using TaqMan® Universal PCR Master Mix on an ABI Prism 7900HT device. Taqman gene and miR assay (Life Technologies, Carlsbad, CA, USA) used were STAT5b (Hs00273500_m1), hsa-miR-150-5p (000473) and hsa-miR-630 (001563). Data were normalized according to GAPDH (Hs99999905_m1) and U6snRNA (001973) levels for gene expression and miR expression data respectively.

### Western blotting

Total protein extraction was performed by homogenization in RIPA lysis buffer (50 mM Tris pH 7.5, 150 mM NaCl, 1% Triton X-100, 1 mM EDTA, 1% sodium deoxycholate and phosphatases 1% cocktail protease inhibitors, 0.5 mM sodium orthovanadate). Lysates were incubated on ice for 30 min and centrifugated at 12,000 g for 20 min at 4°C. Supernatants were then quantified with Bradford Protein Assay (Bio-Rad, Hercules, CA, USA) according to the manufacturer's protocol and boiled in reducing SDS sample buffer (200 mM Tris–HCl [pH 6.8], 40% glycerol, 20% β-mercaptoethanol, 4% sodium dodecyl sulfate, and bromophenol blue); 30 ug of protein lysate was the amount per run for every sample in Criterion™ TGX™ Precast Gels 4-20% (Bio-Rad) and then transferred to Hybond ECL membranes (Amersham, GE HEALTHCARE BioScience, Amersham, UK). Membranes were blocked for 1 h in 5% non-fat dried milk in Tris-buffered saline plus 0.5% Tween 20 (TBS-t) and incubated overnight with the appropriate primary antibody at 4°C. Membranes were then washed in TBS-t and incubated with the appropriate secondary antibody. Both primary and secondary antibodies were diluted in 5% non-fat dried milk in TBS-t. Detection was performed using ECL Western Blotting Detection Reagents or by ECL Plus Western Blotting Detection Reagents (Amersham, GE HEALTHCARE BioScience, Amersham, UK). Antibodies against Stat5 (Stat5a and Stat5b isoforms #9363; Cell Signaling, Danvers, MA, USA), phospho-Stat5 (Tyr694) (#9351; Cell Signaling, Danvers, MA, USA) and β-actin (C4) (sc-47778; Santa Cruz Biotechnology, Inc., Santa Cruz, CA, USA) were used. All secondary antibodies were obtained from Santa Cruz Biotechnology. All the antibodies were used in accordance with the manufacturer's instructions. Images of radiograms were acquired through the HP Precision ScanJet 5300 C Scanner (Hewlett-Packard, Palo Alto, CA, USA).

### Luciferase assay

The pEZX-MT06 target reporter vectors containing full-length wild-type STAT5 3′UTR were purchased from GeneCopoeia. Mutagenized sequence for STAT5b was obtained using QuikChange II Site-Directed Mutagenesis Kit (Agilent Technologies, Santa Clara, CA, USA). Designed Primer sequences are: STAT5b mutagenesis Forward (5′-AATTCCAACCATGAGGATGGG-3′) and STAT5b mutagenesis Reverse (5′-AGTGCCAACTGCAAAGTGA-3′). Twenty-four hours before transfection, 2.5×10^5^ cells were plated in a 6-well plate (each well). Ten pmoles of miR-150-5p mimic or negative control mimic miRNA (ThermoFisher miRIDIAN, Waltham, MA, USA) (Catalogue Number: C-300632-03 and CN00-1000-01 for miR-150-5p and negative control respectively) was transfected into 293T cells together with 2 ug of either wild type or mutated pEZX-MT06 clones using Lipofectamine 2000 according to manufacturer's protocol. Twenty-four hours post-transfection, cells were harvested and seeded again in a 96 well plate in five replicates for each condition. Luciferase assay was performed 48 hours after transfection by the Luc-Pair Duo miR luciferase assay kit (GeneCopoeia, Rockville, MD, USA). Firefly luciferase activity was normalized to renilla activity.

### Generation of lentiviral particles

Third generation lentiviral vectors containing the mature human miR-150-5p sequence were purchased from GeneCopoeia (Rockville, MD, USA) (CatN: LPP-HmiR0306-MR03-050). Production of lentiviral particles was performed as previously described [42]. All viruses were titered (based on percentage of green fluorescein protein (GFP) positive cells) 48 hours after transduction of human fibrosarcoma HT-1080 cells.

### Culture and transduction of primary JMML patient samples

Primary cells were thawed, and dead cells were removed by use of a magnetic column (Miltenyi, Dead-cell removal kit), following the instructions of the manufacturer. Live cells were cultured in StemSpan™ SFEM media (Stem Cell Technologies, Vancouver, BC, Canada) supplemented with 20% heat-deactivated FBS, human GM-CSF (10 ng/ml), human FLT3-Ligand (25 ng/mL), human IL-3 (10 ng/ml), human IL-6 (10 ng/ml), murine SCF (25 ng/mL) and human TPO (10 ng/mL) [42]. All cytokines were purchased from PeproTech (Rocky Hill, NJ, USA). Cells were transduced with lentiviral particles containing either empty control vector or miR-150-5p overexpressing vector. A multiplicity of infection of 10 was used. To facilitate transduction, Polybrene was added to a final concentration of 8 μg/mL. Cells were centrifuged at 1,200G for one hour (at 32°C) and incubated at 37°C overnight. Polybrene-containing media was removed at 24 hours and cells were sorted for GFP positivity and DAPI negativity at 48 hours after transduction. In two JMML patient samples, a fraction of the sorted cells was used for RNA extraction. A Trizol reagent-chloroform extraction method was used, followed by a column-based clean-up of the RNA (Rneasy Micro Kit, Qiagen, Hilden, Germany). RNA was reverse transcribed using the ABI miR reverse transcription kit, as well as miR-150-5p and U44 specific primers. MiR-150-5p expression levels were evaluated by qRT-PCR. Expression levels of U44 were used to normalize the raw miR-150-5p Cq values. GFP-sorted cells were mixed with non-GFP cells at a 4 to 1 ratio. GFP-positivity was evaluated by flow cytometry. The frequency of GFP positive cells in control versus miR-150-5p overexpressing JMML cell populations was calculated for a maximum period of 7 days after sorting the cells.

## SUPPLEMENTARY FIGURE AND TABLES






