# Supplementary material for: MicroRNA fingerprints in juvenile myelomonocytic leukemia (JMML) identified miR-150-5p as a tumor suppressor and potential target for treatment
Source: Oncotarget. 2016 Jul 13;7(34):55395–408. doi: 10.18632/oncotarget.10577 (PMC5342425; doi:10.18632/oncotarget.10577)
Supplement: Supplementary file 1 [file oncotarget-07-55395-s001.pdf]

MicroRNA fingerprints in Juvenile Myelomonocytic Leukemia (JMML) identified miR-150-5p as a tumor suppressor and potential target for treatment

SUPPLEMENTARY FIGURE AND TABLES

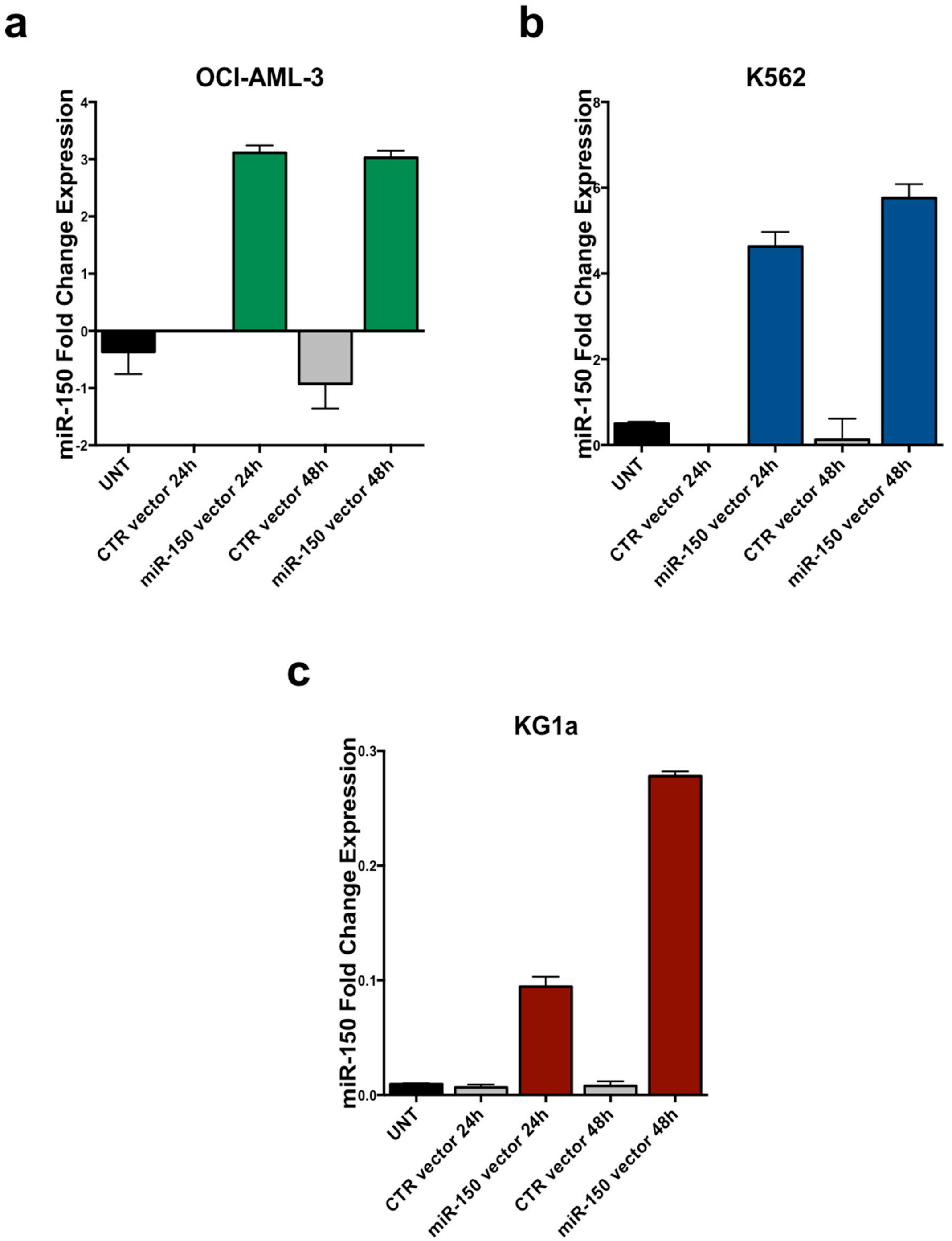

Supplementary Figure S1: MiR-150-5p overexpression in AML cell lines. miR-150-5p expression in OCI-AML-3 **a.**, K562 **b.** and KG1a **c.** cells after transfection with a miR-150-5p precursor overexpressing vector or empty vector (control) as measured by qRT-PCR. Results are expressed as fold change with respect to controls after 24 hours.

Supplementary Table S1: Deregulated microRNAs in *PTPN11*, *KRAS* and *NRAS* subsets.

See Supplementary File 1

Supplementary Table S2: Supplemental Table S2. Complete Nanostring Analyzed microRNAs data.

Complete list of analyzed microRNAs in JMML patients compared to Healthy Donors controls, performed using the nCounter Human v2 miRNA Expression Assay. Accession numbers from miRBase (http://www.mirbase.org) are reported. Statistical analyses were performed using nSolver Analysis Software (nanoString Technologies) and LIMMA package from the Bioconductor R project, as provided by the Carmaweb tool[41]

See Supplementary File 2
