# Supplementary material for: MicroRNA fingerprints in juvenile myelomonocytic leukemia (JMML) identified miR-150-5p as a tumor suppressor and potential target for treatment
Source: Oncotarget. 2016 Jul 13;7(34):55395–408. doi: 10.18632/oncotarget.10577 (PMC5342425; doi:10.18632/oncotarget.10577)
Supplement: Supplementary file 2 [file oncotarget-07-55395-s002.docx]

**Supplemental Table S1. Deregulated microRNAs in *PTPN11*, *KRAS* and *NRAS* subsets**

| **Deregulated microRNAs in *PTPN11* patient subset** | | | | | |  |
| --- | --- | --- | --- | --- | --- | --- |
|  | **Gene Name** | | **Accession #** | | **P-value** | **Fold-Change** |
|  | ***Upregulated*** | |  | |  |  |
|  | hsa-miR-363-3p | | MIMAT0000707 | | 0.027 | 4.21 |
|  | hsa-miR-125b-5p | | MIMAT0000423 | | 0.022 | 3.99 |
|  | hsa-miR-630 | | MIMAT0003299 | | 0.014 | 3.05 |
|  | hsa-miR-224-5p | | MIMAT0000281 | | 0.025 | 2.91 |
|  | hsa-miR-494 | | MIMAT0002816 | | 0.043 | 2.24 |
|  | hsa-miR-548ai | | MIMAT0018989 | | 0.043 | 1.99 |
|  | hsa-miR-181a-5p | | MIMAT0000256 | | 0.025 | 1.92 |
|  | ***Downregulated*** | |  | |  |  |
|  | hsa-miR-148a-3p | | MIMAT0000243 | | <0.001 | -4.33 |
|  | hsa-miR-150-5p | | MIMAT0000451 | | 0.002 | -3.31 |
|  | hsa-miR-342-3p | | MIMAT0000753 | | 0.022 | -2.28 |
|  | hsa-let-7g-5p | | MIMAT0000414 | | 0.018 | -2.17 |
|  |  | |  | |  |  |
| **Deregulated microRNAs in *KRAS* patient subset** | | | | | |  |
|  | **Gene Name** | | **Accession #** | **P-value** | | **Fold-Change** |
|  | ***Upregulated*** | |  |  | |  |
|  | hsa-miR-630 | | MIMAT0003299 | <0.001 | | 7.50 |
|  | hsa-miR-3195 | | MIMAT0015079 | 0.022 | | 3.34 |
|  | hsa-miR-4508 | | MIMAT0019045 | 0.039 | | 3.04 |
|  | hsa-miR-320e | | MIMAT0015072 | 0.009 | | 3.00 |
|  | hsa-miR-377-3p | | MIMAT0000730 | 0.011 | | 2.65 |
|  | hsa-miR-224-5p | | MIMAT0000281 | 0.034 | | 2.51 |
|  | hsa-miR-520e | | MIMAT0002825 | 0.040 | | 2.48 |
|  | hsa-miR-548ai | | MIMAT0018989 | 0.018 | | 2.31 |
|  | hsa-miR-500a-5p | | MIMAT0004773 | 0.018 | | 2.21 |
|  | hsa-miR-130a-3p | | MIMAT0000425 | 0.040 | | 2.14 |
|  | hsa-miR-376c | | MIMAT0000720 | 0.048 | | 2.14 |
|  | hsa-miR-23a-3p | | MIMAT0000078 | 0.014 | | 2.12 |
|  | hsa-miR-340-5p | | MIMAT0004692 | 0.049 | | 1.99 |
|  | hsa-miR-378a-3p | | MIMAT0000732 | 0.032 | | 1.99 |
|  | ***Downregulated*** | |  |  | |  |
|  | hsa-miR-150-5p | | MIMAT0000451 | 0.002 | | -7.63 |
|  | hsa-miR-1260a | | MIMAT0005911 | 0.009 | | -3.05 |
|  | hsa-miR-146b-5p | | MIMAT0002809 | 0.007 | | -2.93 |
|  | hsa-miR-4454 | | MIMAT0018976 | 0.019 | | -2.68 |
|  | hsa-miR-26a-5p | | MIMAT0000082 | 0.005 | | -2.66 |
|  | hsa-miR-29b-3p | | MIMAT0000100 | 0.019 | | -2.59 |
|  | hsa-miR-30b-5p | | MIMAT0000420 | 0.008 | | -2.57 |
|  | hsa-let-7f-5p | | MIMAT0000067 | 0.024 | | -2.35 |
|  | hsa-miR-342-3p | | MIMAT0000753 | 0.033 | | -2.11 |
|  | hsa-miR-106b-5p | | MIMAT0000680 | 0.022 | | -2.08 |
|  | hsa-miR-29a-3p | | MIMAT0000086 | 0.028 | | -2.02 |
|  |  | |  |  | |  |
| **Deregulated microRNAs in *NRAS* patient subset** | | | |  | |  |
|  | **Gene Name** | **Accession #** | | **P-value** | | **Fold-Change** |
|  | ***Upregulated*** |  | |  | |  |
|  | hsa-miR-21-5p | MIMAT0000076 | | 0.035 | | 9.29 |
|  | hsa-miR-143-3p | MIMAT0000435 | | 0.007 | | 7.36 |
|  | hsa-miR-1246 | MIMAT0005898 | | 0.027 | | 6.55 |
|  | hsa-miR-363-3p | MIMAT0000707 | | 0.012 | | 5.07 |
|  | hsa-miR-1283 | MIMAT0005799 | | 0.048 | | 3.44 |
|  | hsa-miR-630 | MIMAT0003299 | | 0.015 | | 2.82 |
|  | ***Downregulated*** |  | |  | |  |
|  | hsa-miR-150-5p | MIMAT0000451 | | 0.010 | | -3.72 |
|  | hsa-miR-1260a | MIMAT0005911 | | 0.011 | | -3.48 |
|  | hsa-miR-720 | MIMAT0005954 | | 0.043 | | -3.46 |
|  | hsa-miR-4454 | MIMAT0018976 | | 0.017 | | -3.38 |
|  | hsa-let-7g-5p | MIMAT0000414 | | 0.007 | | -3.12 |
|  | hsa-let-7d-5p | MIMAT0000065 | | 0.015 | | -3.05 |
|  | hsa-let-7a-5p | MIMAT0000062 | | 0.022 | | -2.97 |
|  | hsa-miR-486-3p | MIMAT0004762 | | 0.019 | | -2.75 |
|  | hsa-let-7b-5p | MIMAT0000063 | | 0.030 | | -2.44 |
|  | hsa-miR-146b-5p | MIMAT0002809 | | 0.043 | | -2.09 |
